# Mechanistic insight into cadmium-induced inactivation of the Bloom protein

**DOI:** 10.1038/srep26225

**Published:** 2016-05-19

**Authors:** Wei Qin, Nicolas Bazeille, Etienne Henry, Bo Zhang, Eric Deprez, Xu-Guang Xi

**Affiliations:** 1 College of Life Sciences, Northwest A&F University, Yangling, Shaanxi 712100, China; 2 LBPA, CNRS UMR8113, IDA FR3242, ENS Cachan, Université Paris-Saclay, 94235 Cachan, France

## Abstract

Cadmium is a toxic metal that inactivates DNA-repair proteins via multiple mechanisms, including zinc substitution. In this study, we investigated the effect of Cd^2+^ on the Bloom protein (BLM), a DNA-repair helicase carrying a zinc-binding domain (ZBD) and playing a critical role to ensure genomic stability. One characteristics of BLM-deficient cells is the elevated rate of sister chromatid exchanges, a phenomenon that is also induced by Cd^2+^. Here, we show that Cd^2+^ strongly inhibits both ATPase and helicase activities of BLM. Cd^2+^ primarily prevents BLM-DNA interaction via its binding to sulfhydryl groups of solvent-exposed cysteine residues and, concomitantly, promotes the formation of large BLM multimers/aggregates. In contrast to previously described Cd^2+^ effects on other zinc-containing DNA-repair proteins, the ZBD appears to play a minor role in the Cd^2+^-mediated inhibition. While the Cd^2+^-dependent formation of inactive multimers and the defect of DNA-binding were fully reversible upon addition of EDTA, the inhibition of the DNA unwinding activity was not counteracted by EDTA, indicating another mechanism of inhibition by Cd^2+^ relative to the targeting of a catalytic residue. Altogether, our results provide new clues for understanding the mechanism behind the ZBD-independent inactivation of BLM by Cd^2+^ leading to accumulation of DNA double-strand breaks.

Bloom’s syndrome (BS) is a rare, autosomal and recessive disease resulting from the mutational inactivation of a human RecQ family helicase encoded by the *blm* gene^1^. BS is characterized by proportional dwarfism, erythema on sun-exposed skin, hyper- or hypo-pigmented skin areas, immunodeficiency and subfertility^2^. Persons with BS have a high predisposition to cancer and increased risk for early-onset type-II diabetes^3^. The *blm* gene encodes BLM, a 1417-amino acids protein containing several conserved motifs including a zinc-binding domain (ZBD). Previous works have shown that mutation of any of the four conserved Cys residues of the ZBD leads to the BS^4^,^5^. Moreover, we have previously shown that the ZBD of RecQ helicases plays a key role in protein folding and is involved in DNA-binding^6^. Thus, alteration of the zinc coordination state and potentially metal-catalyzed oxidation could impair BLM-mediated DNA-repair processing events. In addition to numerous cytological characteristics including high rates of loss of heterozygosity^7^,^8^,^9^, chromosome abnormalities (telomere fusions, ring chromosomes and quadriradial chromosomes^10^), the most striking feature of BLM-deficient cells or cells bearing an impaired BLM mutant is characterized by elevated rates of sister chromatid exchanges (SCEs)^11^. Interestingly, it was shown that Cadmium (Cd) also provoked elevated rates of SCEs in human cell cultures^12^. Thus, the effect of Cd^2+^ on human cell lines shares cytological characters with BLM-deficient cells, establishing a connection between BLM and Cd^2+^.

Cd^2+^ is considered as an important health hazard due to its long retention time and bioaccumulation in human body^13^. Epidemiological and animal experiments have revealed multifactorial carcinogenic properties of cadmium^14^. Exposure to Cd^2+^ is associated with cancers of lung, prostate, pancreas and kidney^15^. Among the various carcinogenic effects of Cd^2+^, DNA damage accumulation due to inhibition of DNA-repair enzymes is considered as one of the major underlying process^16^,^17^. Unlike numerous toxic metal compounds, Cd^2+^ is considered as weakly mutagenic. Nevertheless, Cd^2+^ is known to severely increase the genotoxic effects of various mutagens in mammalian cells, including ionizing radiations and DNA alkylating agents used at low non-cytotoxic concentrations^18^,^19^. Many studies using yeast or human cells suggest that DNA-repair systems represent highly sensitive targets for Cd^2+^. However, the precise mechanism behind carcinogenicity remains to be determined. Although many *in vitro* studies on Cd^2+^-mediated toxic effects have been performed with proteins involved in the Base and Nucleotide Excision Repair (BER/NER)^20^,^21^, Mismatch Repair (MMR)^22^,^23^ and Non-Homologous End-Joining (NHEJ)^24^, it is a difficult task to highlight a general/common mechanism underlying Cd^2+^-mediated inhibition of DNA-repair systems. Nevertheless, it appears that detrimental effects of Cd^2+^ on DNA-repair proteins occur through the binding of Cd^2+^ to functional sulfhydryl groups^23^,^25^, and the replacement of Zn^2+^ by Cd^2+^ in ZBDs represents one cause for protein dysfunctions.

BLM facilitates homologous recombination (HR) between diverged homologous sequences^26^. Among the different DNA-repair systems, HR is remarkable by its ability to accurately repair DNA double-strand breaks. Defects in the HR machinery are often associated with cell cycle deregulations, apoptosis or genomic instability. Until now, HR remains the only DNA-repair pathway for which there is no clear evidence of Cd^2+^-dependent inhibition, although previous studies have shown characteristic features of HR dysfunction following Cd^2+^ uptake such as elevation of SCEs^12^,^15^ and deregulation of the MRE11-dependent pathway that interacts with the HR machinery^24^.

Based on previous observations showing inhibitory effects of Cd^2+^ on zinc-containing DNA-repair proteins and taking into account characteristic phenotypes of Cd^2+^-exposed human and yeast cells^12^, we addressed in the present study the molecular mechanisms of Cd^2+^-dependent BLM inactivation. We demonstrated that Cd^2+^ strongly inhibits both the ATPase and helicase activities of recombinant BLM and that Cd^2+^ primarily prevents BLM-DNA interaction due to the formation of large BLM multimers/aggregates. This formation of large multimers/aggregates is dependent on the protein redox state and is fully reversible by EDTA. Consequently, EDTA rescues the DNA-binding defect in the presence of Cd^2+^. Interestingly, EDTA did not fully counteract the Cd^2+^-dependent inhibition of the proper DNA unwinding activity. Altogether, our results indicate that Cys in the reduced state are primarily targeted by Cd^2+^. Surface and solvent-exposed Cys mediate the Cd^2+^-dependent formation of aggregates (reversible by EDTA) whereas at least one additional residue, playing a key role in the catalytic process, is targeted by Cd^2+^ in an irreversible manner.

## Results

The full-length BLM helicase (BLM^full-length^) is composed of 1417 amino acid and contains the helicase core (BLM^642–1290^) harboring two RecA domains and forming ATP-binding sites, the ZBD (pos. 994–1068) and the winged-helix domain (pos. 1069–1189), whereas the N- and C-terminal domains are more related to protein-protein interactions and nuclear localization^27^,^28^. BLM^642–1290^, used throughout this study together with BLM^full-length^ and *E. coli* RecQ (RecQ^*E.coli*^), displays full activities comparable to BLM^full-length ^27.

### Cadmium severely impairs both helicase and ATPase activities of BLM

**Figure 1:**
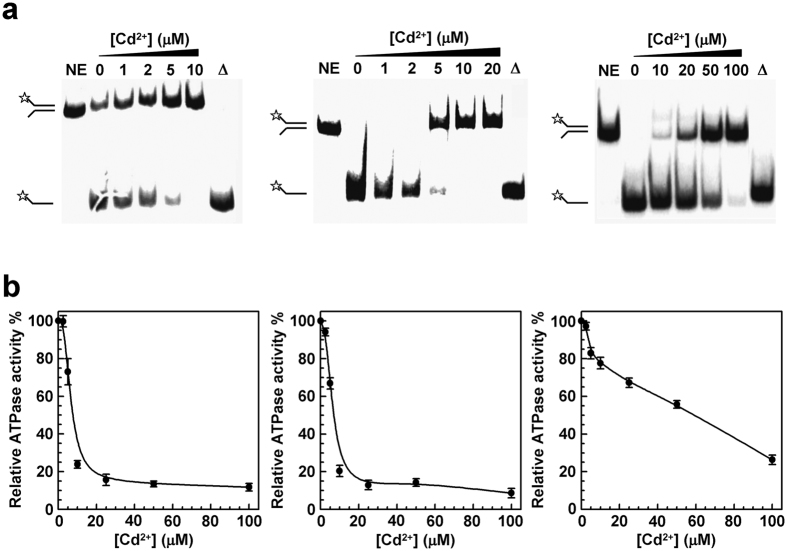
Effect of Cd^2+^ on DNA unwinding (**a**) and ATPase (**b**) activities of BLM^642−1290^ (left), BLM^full-length^ (middle) and RecQ^*E.coli*^ (right). (**a**) DNA helicase activity was revealed by a radioactive assay as described in Methods. 40 nM of proteins were pre-incubated in the presence of increasing concentrations of CdCl_2_ for 2 min at 37 °C. The unwinding reaction was initiated by the addition of the DNA substrate (fork duplex substrate; see Supplementary Table S1) and was carried out for 20 min at 37 °C. NE: no enzyme; ∆: Heat denatured DNA substrate. (**b**) 200 nM of proteins were pre-incubated in the presence of increasing concentrations of CdCl_2_ for 2 min at 37 °C before initiation of the ATPase activity. The ATPase activity was carried out for 10 min at 37 °C (see Methods for more details).

To determine the effect of Cd^2+^ on BLM activities, we first measured the helicase activities of BLM^full-length^ and BLM^642−1290^ in the presence of increasing concentrations of CdCl_2_. DNA unwinding activities of both proteins were strongly inhibited by Cd^2+^ as measured by a radioactive assay: 95% and 100% of inhibition at 5 and 10 μM Cd^2+^, respectively (Fig. 1a). Interestingly, the RecQ^*E.coli*^ unwinding activity was actually inhibited by Cd^2+^ but to a much lesser extent compared to BLM proteins. Indeed, the same inhibition level (>95%) of RecQ^*E.coli*^ was obtained at 100 μM CdCl_2_. These results, suggesting that BLM represents a more sensitive target for Cd^2+^, were confirmed by stopped-flow FRET experiments allowing measurements of the unwinding kinetic rate constant of BLM by using partial duplex DNA labeled with fluorescein and hexachlorofluorescein as a donor and acceptor, respectively^29^,^30^ (Supplementary Fig. S1; see Supplementary Table S1 for details about the structure of the DNA substrate): the levels of Cd^2+^-dependent inhibition of BLM^full-length^ and BLM^642−1290^ helicase activities were similar and significantly higher than that observed with RecQ^*E.coli*^ (≈10-fold higher). Thus, Cd^2+^ displays a selective profile for BLM and most likely targets its helicase core since no significant difference in the Cd^2+^-dependent inhibitions of BLM^full-length^ and BLM^642–1290^ was observed.

We next measured the ATPase activity of the three proteins in the presence of increasing CdCl_2_ concentrations. Again, Cd^2+^ strongly inhibited the ATPase activities of both BLM^full-length^ and BLM^642−1290^, while the ATPase activity of RecQ^*E.coli*^ was much less inhibited (Fig. 1b). The corresponding IC_50_ values (inhibition concentration 50%) determined for BLM^full-length^ and BLM^642−1290^ were 6.7 and 7.3 μM, respectively, whereas the IC_50_ was much higher (65 μM) for RecQ^*E.coli*^ (Table 1). This result closely parallels, at least qualitatively, the one obtained for the inhibition of DNA unwinding activity as explained above.

### Stoichiometry of Cadmium binding to BLM

**Figure 2:**
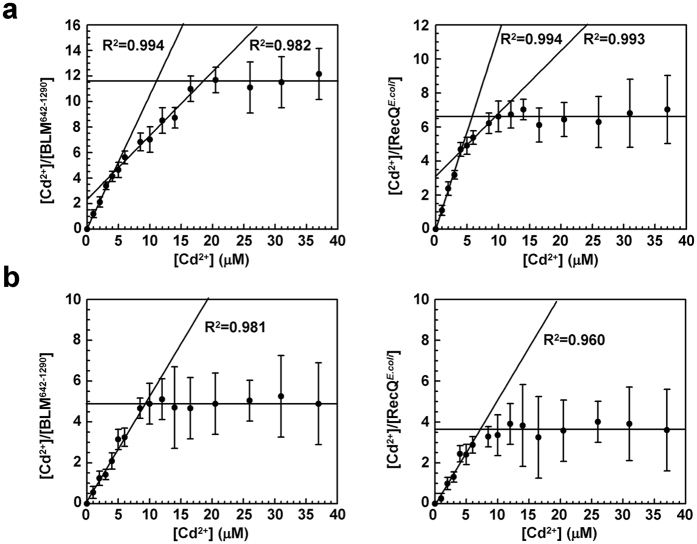
Study of the Cd^2+^:protein stoichiometries for BLM^642−1290^ (left) and RecQ^*E.coli*^ (right) before (**a**) or after (**b**) treatment with 0.5 mM N-Ethylmaleimide (NEM). Increasing concentrations of CdCl_2_ (0–37.5 μM) were added to 0.5 μM of BLM^642−1290^ or RecQ^*E.coli*^ diluted in Tris-HCl buffer (50 mM, pH 8.0) supplemented with 50 mM NaCl and 1 mM DTT. The mixture was further incubated for 5 min at 25 °C. Protein-Cd^2+^ complexes were then discarded using Q-sepharose beads. The Cd^2+^:protein stoichiometries were deduced from the determination of free Cd^2+^ remaining in solution, using the fluorescence-based Measure-iT Cadmium assay as described in Methods.

Before addressing the mechanism of BLM inhibition, we assessed whether BLM is a direct target of Cd^2+^. We then investigated the Cd^2+^:BLM^642−1290^ stoichiometry using a fluorescence-based assay (see Methods). The Cd^2+^:RecQ^*E.coli*^ stoichiometry was studied in parallel for comparison. We found a stoichiometry of 11–12 Cd^2+^ per BLM^642−1290^ molecule (Fig. 2a, left) and a significantly lower stoichiometry was found for RecQ^*E.coli*^: 6–7 Cd^2+^ per protein (Fig. 2a, right). Interestingly, each representation displayed two slopes, reflecting two types of sites characterized by distinct accessibilities and affinities.

Previous studies have reported that free sulfhydryl groups of Cys are good candidates for Cd^2+^ binding^25^. BLM^full-length^ and BLM^642–1290^ contain 30 and 19 Cys, respectively (11 in RecQ^*E.coli*^). To determine whether Cd^2+^ actually targets Cys of BLM^642–1290^ and RecQ^*E.coli*^, stoichiometry experiments were repeated in the presence of N-Ethylmaleimide (NEM), a thiol-alkylating agent that forms stable covalent thioether bonds with sulfhydryls of reduced Cys. NEM treatment significantly altered Cd^2+^:protein stoichiometries with two features: the Cd^2+^:protein stoichiometry was decreased from 11–12 to 5 and from 6–7 to 3–4, for BLM^642–1290^ and RecQ^*E.coli*^, respectively (Fig. 2b). Second, in contrast to experiments performed without NEM, the stoichiometry curves displayed one slope, corresponding to the low-affinity binding site cluster for both proteins. Altogether, the results show that these helicases display at least two types of Cys clusters. Assuming that (i) Cd^2+^ possesses a higher affinity for Cys localized at the surface compared to residues localized in the protein core, (ii) NEM only interacts with surface residues for steric hindrance reason, we conclude that the 1^st^ Cys cluster (surface) is characterized by high affinity for Cd^2+^ without NEM but does not anymore interact with Cd^2+^ in the presence of NEM while the 2^nd^ cluster (protein core) is characterized by weaker affinity for Cd^2+^ due to lower accessibility and is not influenced by NEM. We estimated that the 1^st^ and 2^nd^ clusters are composed by 6–7 and 5 Cys, respectively, in BLM^642–1290^ (3 and 3–4, respectively, in RecQ^*E.coli*^). The remaining Cys that do not interact with Cd^2+^ could be related to residues totally buried in the protein structure. The possible implication of the BLM zinc finger motif is addressed in the following section together with the issue of the Cadmium effect on the proper BLM/DNA interaction.

### Cd^2+^ binding to BLM primarily impairs its DNA-binding activity

To gain further insight into the mechanism of Cd^2+^-induced inhibitions of DNA unwinding and ATPase activities, we examined DNA-binding activities of BLM^full-length^ and BLM^642–1290^ in the presence of increasing CdCl_2_ concentrations by using a steady-state fluorescence anisotropy assay^31^. We first determined DNA-binding isotherms curves (i) in the absence of Cd^2+^ to define the experimental conditions in terms of protein concentrations (*i.e*. high enough above the *K*_d_ to ensure DNA saturation) and (ii) in the presence of Cd^2+^ and absence of any protein to determine the CdCl_2_ concentration range in which the fluorescence anisotropy of the fluorescein-labeled DNA was not influenced by direct Cd^2+^-DNA interactions (data not shown). A protein concentration of 200 nM and CdCl_2_ concentrations up to 100 μM were found to correspond to optimal conditions and then, were used in subsequent experiments.

**Figure 3:**
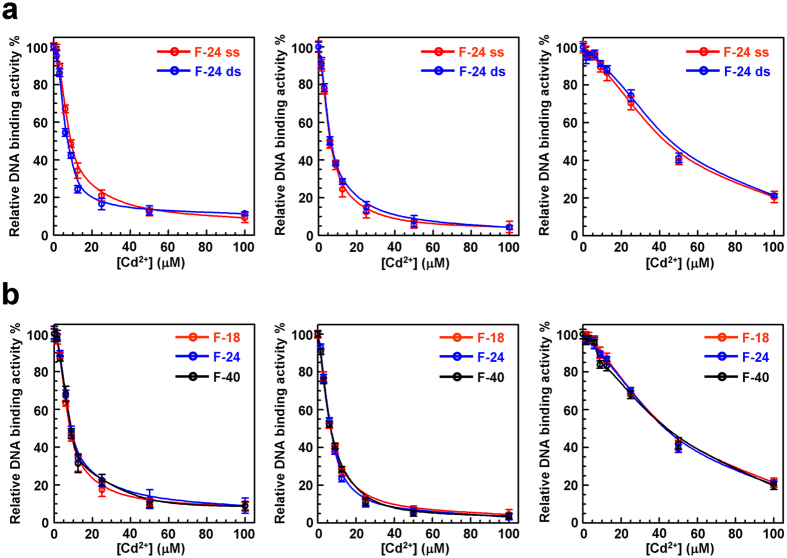
Effect of Cd^2+^ on DNA-binding properties of BLM^642−1290^ (left), BLM^full-length^ (middle) and RecQ ^*E.coli*^ (right). The DNA-binding activity was measured by monitoring the steady-state fluorescence anisotropy of fluorescein-labeled DNA at 25 °C. Increasing concentrations of Cd^2+^ (0–100 μM) were added to 200 nM protein before addition of DNA (5 nM). The relative DNA-binding activity was calculated according to Eq. 2. (**a**) Influence of Cd^2+^ on the binding of helicases to DNA, using single- (ss) or double-stranded (ds) 3′-fluorescein-labeled 24-mer DNA (F-24). (**b**) Influence of Cd^2+^ on the binding of helicases to fluorescein-labeled ssDNA of various lengths: 18-, 24- and 40-mer (F-18, F-24 and F-40, respectively). The sequences of oligonucleotides are described in Supplementary Table S1 and corresponding IC_50_ values are reported in Table 1.

BLM^full-length^ and BLM^642−1290^ displayed similar inhibition profiles of the DNA-binding activities (Fig. 3). The Cd^2+^-dependent inhibitions of the protein-DNA interaction were efficient, with IC_50_ values in the 6.6–10.2 μM range (Table 1). These values were consistent with those derived from the inhibition of helicase and ATPase activities. Moreover, the inhibition profiles were similar, regardless of the nature of the DNA substrate, single- (ss) or double-stranded (ds) DNA (Fig. 3a) or the DNA length, from 18- to 40-mer (Fig. 3b). However, the RecQ^*E.coli*^ DNA-binding activity was weakly affected by Cd^2+^ (IC_50_ = 39–44 μM), as observed for its ATPase or helicase activity, confirming that BLM is more sensitive to Cd^2+^ than RecQ^*E.coli*^.

The binding of BLM^642–1290^ to DNA was next studied in the presence of Cd^2+^ and various amino-acids such as Cys, His and Val. Among the different amino-acids, Cys is the residue forming by far the most stable complex^32^. Accordingly, when amino-acids were pre-incubated with both Cd^2+^/DNA before addition of the protein, the BLM^642–1290^ DNA-binding activity was fully restored by Cys but only partially restored by His or Val (Supplementary Fig. S2). The counteracting effect of Cys was also observed on the ATPase activity of BLM^642–1290^ (data not shown). By contrast, the addition of amino-acids after pre-incubation of BLM^642–1290^ with Cd^2+^/DNA did not restore the DNA-binding activity, regardless of the nature of the amino-acid (Supplementary Fig. S2). The absence of protective/competition effect by Cys in the latter case can be ascribed to irreversible binding of Cd^2+^ to BLM sulfydryl groups and EDTA only was able to reverse the Cd^2+^-mediated inhibition of DNA-binding (see below).

Taking into consideration that BLM contains a zinc finger that is composed of four conserved Cys^5^, we next investigated whether the BLM ZBD could be a target site for Cd^2+^. Mutation of any one of these Cys leads to the BS and inactivates BLM activity both *in vitro* and *in vivo*^6^. First, we wondered whether Zn^2+^ and Cd^2+^ would be able to bind to the same site and whether the replacement of Zn^2+^ by Cd^2+^ would lead to a conformational change of the BLM ZBD and, consequently, impairs BLM activity. To further investigate the mechanism of Cd^2+^-mediated inhibition and the interplay between Cd^2+^ and Zn^2+^, the influences of both metals on the DNA-binding step were compared using the fluorescence anisotropy-based assay. Interestingly, Cd^2+^ but also Zn^2+^ inhibited the DNA-binding step of BLM^full-length^, BLM^642–1290^ and RecQ^*E.coli*^, however to different extents (Supplementary Fig. S3). Up to 50 μM, Cd^2+^ alone was consistently more potent than Zn^2+^ alone for inhibition. Beyond 50 μM, both metals inhibited BLM^full-length^ and BLM^642–1290^ in a similar manner. To note, no inhibition was observed for BLM^642–1290^ at low Zn^2+^ concentrations (<15 μM). In this concentration range, the inhibitory effect of Cd^2+^ was not reversed by addition of Zn^2+^. The Cd^2+^/Zn^2+^ combination was even more efficient for inhibiting DNA-binding activity than Cd^2+^ alone, a typical synergistic inhibition phenomenon. Such a behavior was also observed with BLM^full-length^, except that low Zn^2+^ concentrations were more efficient for inhibiting the DNA-binding step of BLM^full-length^ compared with BLM^642–1290^. Regarding RecQ^*E.coli*^ DNA-binding activity which was only partially inhibited by Zn^2+^ alone (85% of activity at 100 μM), the more potent effect of Cd^2+^ was not reversed by addition of Zn^2+^. Altogether, these results indicate that, under our experimental conditions, Cd^2+^ and Zn^2+^ do not bind competitively to the same site. It is important to note that, especially with Cys_4_ ZBD (corresponding to BLM or RecQ^*E.coli*^ ZBDs), Cd^2+^ forms much more stable complexes than zinc^33^. Taking into account that a large excess of Zn^2+^ over Cd^2+^ could be required for efficient competition (a condition which is not compatible with the proper Zn^2+^-dependent inhibition described above), we cannot definitively confirm or ruled out a targeting of the BLM ZBD by Cd^2+^. However, it is unlikely that the ZBD alone accounts for the Cd^2+^-dependent inhibition since BLM and RecQ^*E.coli*^ are characterized by different susceptibilities to Cd^2+^; this differential susceptibility appears to be more related to the larger number of targeted Cys in the case of BLM, as suggested by stoichiometry experiments. The relationship between the defect of DNA-binding and the binding of Cd^2+^ to surface Cys residues was further investigated below by studying the Cd^2+^ effect on the multimeric status of BLM.

### Cd^2+^ induces BLM oligomerization

**Figure 4:**
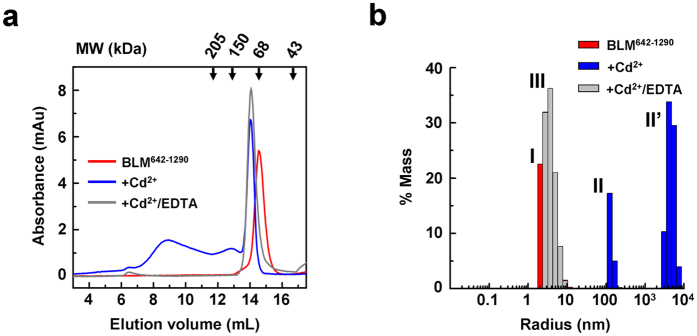
Size distributions of BLM^642–1290^ as measured by size-exclusion chromatography (**a**) and dynamic light scattering (DLS) (**b**) and influence of Cd^2+^. (**a**) Size-exclusion chromatography was performed as described in Methods. The different experimental conditions are explicitly mentioned in the figure (the concentrations of CdCl_2_ and EDTA were 50 μM and 2 mM, respectively). The elution profile corresponds to the absorbance at 280 nm. Molecular weights (MW) used for the calibration are indicated on the top axis. (**b**) Size distribution (in % mass) characterizing BLM^642–1290^ (1.5 μM) in the absence and in the presence of 200 μM CdCl_2_. BLM^642–1290^ alone is characterized by peak I. BLM^642–1290^ in the presence of Cd^2+^ is characterized by two peaks (II+II’). BLM^642–1290^ in the presence of 200 μM CdCl_2_ + 2 mM EDTA is characterized by peak III.

During the course of our experiments, we observed that high Cd^2+^ concentrations (typically >30 μM) induced BLM precipitation in samples. Size-exclusion chromatography analysis showed that while BLM^642–1290^ alone was eluted as a monomer as previously reported^34^, its elution profiles when pretreated with Cd^2+^ displayed three peaks, corresponding to molecular weights of 75, 150 and >205 kDa, respectively (Fig. 4a), indicating that Cd^2+^ induced protein oligomerization. We further studied the oligomeric status of BLM in the absence and presence of Cd^2+^ by Dynamic Light Scattering (DLS) (Fig. 4b). DLS analysis confirmed size-exclusion chromatography experiments. Indeed, without Cd^2+^, BLM^642–1290^ was monodisperse in solution and characterized by a radius of 3.44 nm, compatible with a monomeric form (peak I). The addition of Cd^2+^ dramatically modified the profile of size distribution. The peak I disappeared and was replaced by two peaks (II+II’) corresponding to large BLM multimers, probably non-specific cross-linked aggregates (with MW of 3.2 × 10^5^ and 1.3 × 10^9^ kDa, respectively). Addition of EDTA on Cd^2+^-treated samples of BLM^642–1290^ dissociated higher-order oligomers leading to a recovery of the monomeric form as shown by size-exclusion chromatography (Fig. 4a) or DLS (Fig. 4b; peak III, radius 3.95 nm). Altogether, these results show that Cd^2+^ induces the formation of higher-order BLM oligomers that is reversible upon addition of EDTA.

### Dual effect of DTT on the modulation of the BLM DNA-binding activity by Cd^2+^ and reversibility with EDTA

**Figure 5:**
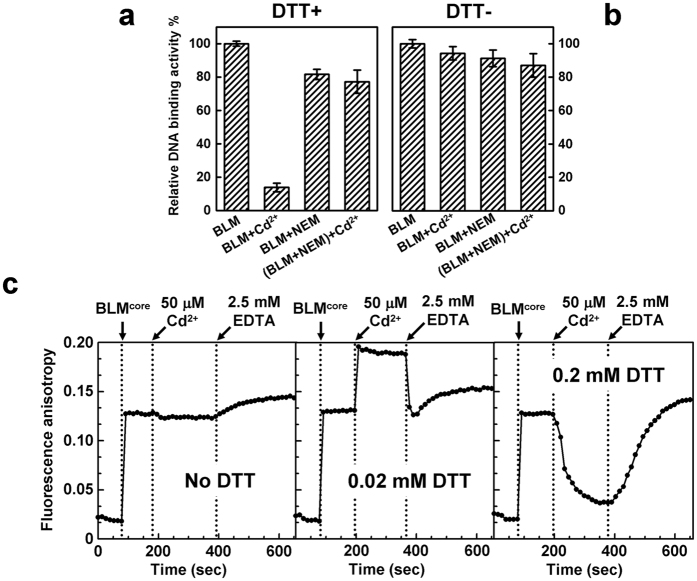
Influence of DTT on the Cd^2+^-mediated inhibition of DNA-BLM^642–1290^ interaction and protective effect of NEM. BLM^642–1290^/DNA complexes were formed using 200 nM protein and 5 nM F-18 oligonucleotide in the presence (**a**) or absence (**b**) of 2 mM DTT. The concentration of CdCl_2_ and NEM were 50 μM and 0.5 mM, respectively. The fluorescence anisotropy was monitored at 25 °C after addition of F-18. The relative DNA-binding activity was calculated according to Eq. 2. (c) Differential effect of Cd^2+^ on the DNA-binding activity of BLM^642–1290^ as a function of DTT concentration and reversibility with EDTA. The time of addition for Cd^2+^/EDTA is explicitly indicated by arrows. Protein and F-18 concentrations were 200 and 5 nM, respectively. The fluorescence anisotropy was monitored at 25 °C as a function of time. Left: no DTT; middle: 0.02 mM DTT; right: 0.2 mM DTT.

DTT has been previously described either as a stimulating or a counteracting agent for Cd^2+^-mediated inhibition^35^,^36^. All the above-mentioned results were obtained under reducing conditions, *i.e*. in the presence of DTT. We then compared the Cd^2+^ effect on the BLM^642–1290^-DNA interaction under reducing and non-reducing conditions (Fig. 5). In the presence of DTT, Cd^2+^ efficiently inhibited the binding of BLM^642–1290^ to DNA (Fig. 5a), in accordance with results shown in Fig. 3. To note, NEM that did not lead to significant effect on its own on the BLM^642–1290^ DNA-binding activity, fully counteracted the inhibitory effect of Cd^2+^, suggesting that Cys of the 1^st^ cluster (surface residues as defined above) mainly mediate the Cd^2+^-dependent inhibition.

By contrast, under non-reducing conditions, Cd^2+^ did not significantly inhibit the BLM^642–1290^ DNA-binding activity (Fig. 5b), suggesting that Cd^2+^-targeted Cys are most likely involved in intra- or inter-molecular disulfide bonds and react with Cd^2+^ upon reduction only (in the form of sulfhydryl groups). The effect of Cd^2+^ on the BLM^642–1290^ DNA-binding activity was further studied under different reducing conditions by varying the DTT concentration. At 0.2 mM DTT (Fig. 5c, right), this effect was comparable to the one previously observed at 2 mM DTT (Fig. 5a), *i.e*. DTT promoted the Cd^2+^-mediated inhibition of protein-DNA interaction, while no Cd^2+^ effect was observed in the absence of DTT (Fig. 5c, left). Interestingly, under limited reducing conditions (0.02 mM DTT; Fig. 5c, middle), the addition of Cd^2+^ led to an increase in the steady-state fluorescence anisotropy, instead of a decrease. This dual effect of DTT remains unclear. We can hypothesize that Cd^2+^ differentially modulates self-assembly properties of BLM^642–1290^ depending on the DTT concentration. As shown above, no free sulfhydryl of surface Cys is available for Cd^2+^ binding in the absence of DTT and the presence of intra/inter-molecular disulfide bonds is compatible with DNA-binding. Under moderate DTT conditions (0.02 mM DTT), a part of Cys residues only could be in their reduced forms and Cd^2+^ could promote the formation of higher-order BLM^642–1290^ multimers of moderate sizes which remain compatible with DNA-binding but leading to higher steady-state anisotropy values due to the size of protein-DNA complexes (larger compared with the size of complexes obtained in the absence of DTT). By contrast, under strong reducing conditions (>0.2 mM DTT), most of surface Cys residues should be in the reduced state and thus, Cd^2+^ promotes large multimers/aggregates, not competent for DNA-binding. In other words, the DNA-binding site should be completely hidden in the context of these large BLM multimers/aggregates. Note that both Cd^2+^ effects (inhibition or stimulation) were abolished by addition of EDTA (Fig. 5c) in a dose-dependent manner (Supplementary Fig. S4). Consistent with our hypothesis, EDTA was shown to reverse Cd^2+^-induced multimers by size-exclusion chromatography and DLS (Fig. 4).

### Cd^2+^-induced inhibition of BLM DNA unwinding activity was not reversed by addition of EDTA

**Figure 6:**
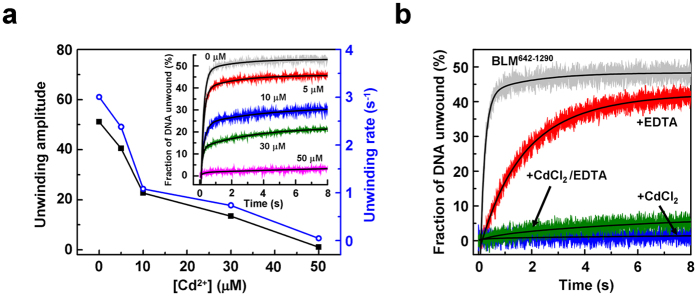
Cd^2+^-induced impairment of helicase unwinding activity is irreversible. (**a**) Dependence of the kinetic rate constant and reaction amplitude as a function of CdCl_2_ concentration as measured by stopped-flow FRET assay. BLM^642–1290^ was first preincubated with varying concentrations of CdCl_2_ for 5 min at 25 °C. The DNA substrate (16-bp duplex with a 20-nt 3′ tail) was then added into the reaction mixture and the reaction was initiated by rapid mixing with 1 mM ATP. Insert: typical kinetics for DNA unwinding in the presence of various CdCl_2_ concentrations. (**b**) EDTA failed to restore Cd^2+^-induced DNA unwinding. BLM^642–1290^ was first preincubated for 5 min at 25 °C in the absence or presence of 50 μM CdCl_2_. Each sample was further incubated for 5 min in the absence or presence of 2.5 mM EDTA, before addition of the DNA substrate and reaction initiation with ATP as explained in the legend of panel (**a**). BLM^642–1290^ alone (grey trace); BLM^642–1290^ + EDTA (red trace); BLM^642–1290^ + Cd^2+^ (blue trace); BLM^642–1290^ + Cd^2+^ + EDTA (green trace). Reactions were performed with 4 nM DNA and 60 nM protein in Tris-HCl buffer (25 mM, pH 7.5) supplemented with 50 mM NaCl, 2 mM MgCl_2_ and 1 mM DTT at 37 °C.

We next addressed the question of whether EDTA could restore the unwinding activity of BLM as measured in the presence of Cd^2+^ using the stopped-flow FRET assay. First, Cd^2+^ significantly reduced both the unwinding kinetic rate constant and the corresponding reaction amplitude characterizing BLM^642–1290^ (Fig. 6a), in accordance with results shown in Fig. 1. Second, we tested the effect of EDTA on the unwinding activity of BLM^642–1290^ *per se* since the Mg^2+^ cofactor is required for this activity. As shown in Fig. 6b, although the kinetic rate constant was lower in the presence of EDTA, the reaction amplitude was only slightly affected. This result shows that BLM^642–1290^ sustains a significant DNA unwinding activity even in the presence of EDTA. Nevertheless, the Cd^2+^-dependent inhibition of BLM activity was not counteracted by EDTA (Fig. 6b), in contrast to that previously observed for the DNA-binding step. The fact that the helicase activity of BLM^642–1290^ cannot be recovered upon addition of EDTA, suggests that Cd^2+^ also inactivates (at least) one additional Cys or another residue, most likely playing a proper catalytic role for DNA unwinding, in an irreversible manner.

## Discussion

The BLM helicase plays key roles in numerous cellular processes including DNA double-strand break repair (DSB), Holliday junction resolution and chromosome segregation^37^,^38^,^39^. In this context, the study of the toxic effect of Cd on BLM is of particular interest for understanding the pivotal role of BLM in keeping genome stability. Among Cd^2+^, Zn^2+^ and Hg^2+^, only Cd^2+^ has been reported to induce SCEs, a unique cytological feature of BLM-deficient cells^40^. Furthermore, recent studies highlight that Cd^2+^ targets major players of the DNA-repair machinery including proteins involved in the BER, NER or MMR pathways^21^,^22^,^23^, although little is known about Cd^2+^ effects on proteins involved in the DSB pathway such as BLM. Here, we investigated the interplay between BLM and Cd^2+^ at the molecular level. We found two distinct molecular mechanisms accounting for the Cd^2+^-mediated inactivation of BLM. Cd^2+^ targets surface Cys in their reduced state and promotes the formation of large BLM multimers and then inhibition of the BLM-DNA interaction. The Cd^2+^-dependent multimerization and DNA-binding inhibition processes were found to be fully reversible upon addition of EDTA. However, the inhibition of the BLM helicase activity was irreversible suggesting another mechanism at the catalytic level, mediated by the targeting of a catalytic residue.

We found that Cd^2+^ was able to efficiently inhibit both helicase and ATPase activities of BLM^full-length^ and BLM^642–1290^ in the low micromolar concentration range. The corresponding IC_50_ values were compatible with values derived from DNA-binding inhibition curves suggesting that the Cd^2+^-dependent inhibition of helicase and ATPase activities could be explained in part by inhibition of the DNA-binding step. The reversibility of the DNA-binding inhibition process by EDTA indicates that the Cd^2+^-dependent inhibition mechanism is not associated with an irreversible structural modification of the protein that could affect DNA-binding properties. It is important to note that, under experimental conditions where Cd^2+^ was maintained below 100 μM, fluorescence anisotropy experiments did not show any significant direct DNA-Cd^2+^ interaction, in accordance with previous studies^41^. Furthermore, we found that Cd^2+^ inhibits both BLM and RecQ^*E.coli*^, however to different extents, with RecQ^*E.coli*^ much less susceptible to Cd^2+^ (≈one order of magnitude) than BLM. This differential susceptibility reinforces the idea that Cd^2+^ dose not directly target DNA in our activity or DNA-binding assays. Instead, this differential susceptibility appears to be related to the number of solvent-exposed Cys contained in the protein structure with RecQ^*E.coli*^ having much less surface Cys (*i.e*. protected by NEM) than BLM^642–1290^ (Fig. 2).

We also tested whether Cd^2+^ could replace Zn^2+^ in the ZBD based on several statements: (i) Cd^2+^ has been previously reported to react with thiol groups, particularly with Cys and glutathione that act as the first line of defense against Cd^2+^ in cells^36^,^42^. (ii) The antagonistic effect of Zn^2+^ on Cd^2+^ has long been documented and previous studies have shown that Cd^2+^-targeted sites in proteins correspond to zinc finger motifs and that the replacement of Zn^2+^ by Cd^2+^ may be reversible^43^,^44^. However, here, we failed to demonstrate that Zn^2+^ prevents the inhibitory effect of Cd^2+^ on BLM. Taking into account the higher affinity of Cd^2+^ over Zn^2+^ for Cys_4_ ZBD, large excess of Zn^2+^ should be required to observe a protective effect against Cd^2+^; it was not possible to satisfy this condition since, instead to rescue BLM DNA-binding activity, Zn^2+^ on its own displayed a significant inhibitory effect although this effect was less important than Cd^2+^. If Cd^2+^ target the helicase ZBD, this is probably not the predominant effect for the following reasons: (i) BLM and RecQ^*E.coli*^ are differentially affected by Cd^2+^, in accordance with their respective number of solvent-exposed Cys (Fig. 2 and Supplementary Table S2) and (ii) we have previously shown that Zn^2+^-coordination to BLM or RecQ^*E.coli*^ ZBD is strictly required for correct protein folding (during production) but dispensable after for both DNA-binding and helicase activities^6^,^34^. The mechanism by which Zn^2+^ inhibits BLM remains unknown and it appears that Zn^2+^ differentially affects helicases of the RecQ family with Zn^2+^ having only a slight inhibition effect on the binding of RecQ^*E.coli*^ to DNA (Supplementary Fig. S3). Consistent with our study on human BLM, it was previously shown that Zn^2+^ also significantly impairs the helicase activity of BLM yeast homologue, Sgs1^45^. It has also been shown that Zn^2+^ enhances the 3′ → 5′ exonuclease activity of another human RecQ helicase, the Werner protein, at the expense of the helicase activity^46^,^47^,^48^.

It was previously shown that the binding of BLM and RecQ^*E.coli*^ to DNA relies on similar mechanisms. Regarding their binding to dsDNA and ssDNA, both proteins involve distinct protein domains with ssDNA-binding sites located along the A1, A2, WH and HRDC domains, whereas the dsDNA-binding site is located near the ZBD^5^,^34^,^49^. Although different between BLM and RecQ^*E.coli*^, the IC_50_ values characterizing protein-DNA interaction inhibitions were similar between ss and dsDNA, reinforcing the idea that the Cd^2+^-dependent DNA-binding inhibition is not strictly related to the ZBD and probably occurs via a more general and common mechanism, regardless of the nature of DNA (ss or ds). Besides the targeting of ZBD, other mechanisms of Cd^2+^-mediated protein inhibitions involved in DNA-repair have been proposed. It was proposed that Cd^2+^ inhibits mismatch repair pathway by abrogating the ATPase activity of the MSH2-MSH6 complex, via a mechanism in which Cd^2+^ binds in a non-specific manner, leading to a stoichiometry of more than hundred Cd^2+^ per protein^50^. In the case of BLM, the number of target sites appears to be limited. In contrast to MSH2-MSH6 complexes that possess a high content of non-specific Cd^2+^ binding sites, we found that BLM^642–1290^ is characterized by a much lower stoichiometry (11–12 Cd^2+^ per protein) and Cys sulfydryl groups are directly involved in this Cd^2+^-binding process. We have characterized two subclasses of Cys targeted by Cd^2+^, based on (i) the presence of two distinct slopes in stoichiometry plots, most likely accounting for differences in accessibility/affinity and (ii) the NEM protective effect. The first class (6–7 residues) is composed by Cys displaying high affinity for Cd^2+^, most likely located at the protein surface while the second class (5 residues) corresponds to Cys with lower affinity for Cd^2+^ and probably buried and located in the protein core.

Our data suggest that the first class of Cys is fully responsible for the Cd^2+^-dependent inhibition of BLM DNA-binding since NEM, which prevents the binding of Cd^2+^ to Cys belonging to this class only, fully rescues the DNA-binding activity in the presence of Cd^2+^ (Fig. 5). The Cd^2+^-mediated inhibition of DNA-binding was strictly dependent on the presence of DTT suggesting the involvement of free sulfydryl groups of surface Cys in the binding of Cd^2+^. Most of the class 1 Cys should be engaged in disulfide bridges under non-reducing conditions, explaining the absence of any inhibitory effect of Cd^2+^ in the absence of DTT. In parallel, we found that Cd^2+^ promotes BLM higher-order multimers or aggregates starting from monodisperse samples of monomers. The mechanism behind at the molecular level is not yet clearly understood and still under investigation. This phenomenon was fully reversed by EDTA, in accordance with the counteracting effect of EDTA on BLM DNA-binding activity. To note, only reducing conditions (to ensure free sulfydryl groups) were compatible with the observation of Cd^2+^-dependent inhibition, suggesting that DNA-binding sites of BLM protomers should be hidden upon the formation of these large non-specific multimers/aggregates. Interestingly, mild reducing conditions, which modulate the number of free -SH at the protein surface, led to an intermediary result between reducing condition (inhibition of DNA-binding by Cd^2+^ as measured by a decrease in the anisotropy value) and non-reducing condition (no effect of Cd^2+^ on the anisotropy value) (Fig. 5c): in this mild reducing condition, Cd^2+^ led to an increase in the anisotropy value, probably accounting for DNA-binding of organized multimers of moderate sizes. Their oligomeric status should be intermediary between monomers and large non-specific multimers/aggregates, with remaining solvent accessible DNA-binding sites. The different number of solvent-exposed Cys between BLM^642–1290^ and RecQ^*E.coli*^, 6–7 and 3, respectively, highlights the relationship between the number of Cys potentially targeted by Cd^2+^ and the extent of the Cd^2+^-dependent DNA-binding inhibition. To note, these numbers of solvent-exposed Cys as determined experimentally agree well with the number of surface Cys based on 3D structures (7 and 3, respectively, without taking into account ZBD Cys; Supplementary Table S2). Nevertheless, the Cd^2+^-dependent inhibition of the BLM unwinding activity was not reversed by EDTA, suggesting an additional catalytic mechanism of inhibition, unrelated to the DNA-binding step. According to the three-dimensional BLM structure^51^,^52^, we performed single point mutations of Cys residues implicated in DNA binding/unwinding (C895S and C901S). The two BLM mutants displayed modest reduced DNA-unwinding activity and their responses to Cd^2+^ were similar to the wild-type BLM (data not shown). This suggests that distinct residues (Cys or other amino-acids) or, alternatively, a combination of the two above-mentioned Cys could be responsible for the irreversible BLM inactivation by Cd^2+^. The underlying mechanism is currently under investigation.

## Methods

### Chemical reagents

CdCl_2_, ZnCl_2_, EDTA, DTT (dithiothreitol), NEM (N-Ethylmaleimide), ATP and Triton X-100 were purchased from Sigma. [γ-^32^P] ATP was purchased from Perkin Elmer.

### Recombinant proteins

RecQ^*E.coli*^ and BLM^642–1290^ helicases were expressed and purified as previously described^34^,^53^. BLM^full-length^ was expressed in *Saccharomyces cerevisiae* JEL-1 strain as previously described^54^ with some modifications in the purification protocol. Briefly, cells were thawed at 4 °C and cell disruption was performed using a French press in a Tris-HCl buffer (50 mM, pH 7.5) supplemented with 500 mM KCl, 10% sucrose, 1 mM DTT and protease inhibitors cocktail (Roche). After DNA fragmentation by sonication, the crude extract was subjected to centrifugation at 30,000 g for 45 min using a SS34 rotor (Sorvall). Filtered supernatant (using 0.45 μm filters) was loaded in 10 mL of Ni^2+^-NTA agarose resin (Qiagen). Beads were washed with 100 mL of K_2_HPO_4_/KH_2_PO_4_ buffer (20 mM, pH 7.4), 0.05% Triton X-100, 10% glycerol, 1 mM DTT (=buffer A-20 mM) supplemented with 500 mM KCl and 20 mM imidazole, followed by 100 mL of buffer A-20 mM supplemented with 500 mM KCl and 50 mM imidazole. Proteins were then eluted with 100 mL of buffer A-20 mM supplemented with 500 mM KCl and 300 mM imidazole. Fractions containing BLM were pooled and directly loaded onto a 10-mL Biogel CHT hydroxyapatite column (Bio-Rad). The column was washed with 100 mL of buffer A-20 mM, followed by 100 mL of buffer A-100 mM. Elution was done with 100 mL of buffer A-350 mM supplemented with 50 mM KCl. Fractions containing BLM were pooled and diluted in 25 mL of buffer A-20 mM supplemented with 50 mM KCl and loaded onto 5 mL of Q-sepharose fast Flow (GE Healthcare). Beads were washed with 100 mL of buffer A-20 mM supplemented with 50 mM KCl, followed by 100 mL of buffer A-20 mM supplemented with 230 mM KCl. BLM was eluted using buffer A-20 mM supplemented with 500 mM KCl. Fractions containing BLM were then concentrated before loading onto a S-200 Superdex HR 10/30 gel filtration column (GE Healthcare) equilibrated in buffer A-20 mM supplemented with 500 mM KCl. Fractions containing BLM were concentrated, dialyzed against 50 mM Tris-HCl, pH 7.5, 100 mM KCl, 0.05% Triton X-100, 1 mM DTT, 25% glycerol and stored at −80 °C. RecQ^*E.coli*^, BLM^642–1290^ and BLM^full-length^ proteins were >95% pure as judged by SDS-PAGE analysis and Coomassie staining (Supplementary Fig. S5).

### Oligonucleotides

The sequences of DNA substrates used for enzymatic or DNA-binding assays are shown in Supplementary Table S1. PAGE-purified fluorescein-labeled or unlabeled synthetic oligonucleotides were purchased from Eurogentec. Double-stranded DNA substrates were obtained by mixing equimolar amounts of complementary strands in 20 mM Tris-HCl, pH 7.5, 100 mM NaCl. The mixture was heated to 95 °C for 5 min and annealing was allowed by slow cooling to room temperature.

### Cadmium binding assay

Cd^2+^ binding to helicases was assayed by incubating BLM^642–1290^ or RecQ^*E.coli*^ (0.5 μM) with increasing concentrations of CdCl_2_ (from 0 to 37.5 μM) in 50 μl of Tris-HCl buffer (50 mM, pH 8.0) supplemented with 50 mM NaCl and 1 mM DTT, for 5 min at 25 °C. This condition is suitable to measure Cd:protein stoichiometries since both protein and Cd^2+^ concentrations were much higher than *K*_d_ values characterizing Cd·Cys complexes^32^. When required, the concentration of NEM was 0.5 mM. Free Cd^2+^ in solution was measured by a fluorescence-based Measure-iT Cadmium assay (Invitrogen) according to the manufacturer’s protocol. To avoid any bias in the measurement of free Cd^2+^ due to equilibrium displacement (Cd^2+^-protein -> Cd^2+^-sensor), proteins were eliminated from the mixture by using Q-sepharose beads before the measurement. Free Cd^2+^ concentrations were deduced from calibration plots using CdCl_2_ solutions of known concentrations (we checked that interaction between free Cd and beads was negligible: fluorescence intensity values obtained after incubation of CdCl_2_ solutions with beads were 95–100% of intensities measured with “input” solutions). The number of protein-bound Cd^2+^ was estimated by subtracting the amount of free Cd^2+^ to the total amount of Cd^2+^.

### Size-exclusion chromatography and dynamic light scattering (DLS) experiments

The size-exclusion chromatography experiment was performed according to Xu *et al*.^55^. Briefly, the chromatography was performed at 25 °C, using an FPLC system (GE healthcare), on a Superdex 200 (analytical grade) column equilibrated with buffer S (20 mM Tris-HCl, pH 7.4, 500 mM NaCl, 1 mM DTT and 5% glycerol (v/v)) +/− 2 mM EDTA. 20 μl of untreated or Cd^2+^-treated BLM (+/− 2 mM EDTA) was loaded on the column (typically in the 2–6 μM concentration range) and was eluted with buffer S +/− 2 mM EDTA, at a flow rate of 0.4 ml/min; the absorbance was continuously monitored at 280 and 260 nm. The standard molecular markers (Sigma) used for calibration were eluted under identical experimental conditions.

DLS measurements were performed using a DynaPro NanoStar instrument (Wyatt Technology, France) equipped with a thermostated cell holder using filtered (0.1 μm filters) solutions in disposable cuvettes (UVette, Eppendorf). The protein concentration was 1.5 μM in a Tris-HCl buffer (50 mM, pH 8.0, 200 mM NaCl, 1 mM DTT) (total volume, 50 μl). The scattered light was collected at an angle of 90°. Recording times were typically between 3–5 min (20–30 cycles in average of 10s each). The analysis was performed with the Dynamics 7.0 software using regularization methods (Wyatt Technology, France). The molecular weight was calculated from the hydrodynamic radius using the following empirical equation 1:


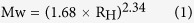


where Mw and R_H_ represent the molecular weight (kDa) and the hydrodynamic radius (nm), respectively.

### ATPase activity assay

The ATPase activity was assayed by measuring the release of free phosphate during ATP hydrolysis^34^. The reaction was carried out for 10 min at 37 °C in an ATPase reaction buffer (Tris-HCl 50 mM, pH 8.0) supplemented with 50 mM NaCl, 3 mM MgCl_2_, 0.1 μg/ml BSA and 1 mM DTT. The reaction was initiated by addition of helicase (200 nM) into a reaction mixture containing 3 μM DNA (25-nt ssDNA) and 2 mM [γ-^32^P] ATP, in the absence or presence of increasing CdCl_2_ concentrations (final volume, 50 μl). The reaction was stopped by transferring 35 μl of the reaction mixture into a hydrochloric solution of ammonium molybdate. The liberated radioactive γ^32^Pi was extracted with a solution of 2-butanol-benzene-acetoneammonium molybdate (750:750:15:1) saturated with water. A volume of 400 μl was removed from the organic phase and radioactivity was quantified using a liquid scintillation counter (Beckman LS 5000CE).

### Helicase assay

1) Radioactive assay: DNA helicase reaction was carried out at 37 °C in a reaction mixture containing 25 mM Tris-HCl, pH 8.0, 50 mM NaCl, 3 mM MgCl_2_, 0.1 μg/ml BSA, 1 mM DTT, 2 mM ATP. To address the Cd^2+^ effect on helicase activity, helicases were preincubated without or with Cd^2+^ for 2 min at 37 °C. The unwinding reaction was initiated by addition of 10fmol of the ^32^P-labeled partial duplex DNA substrate (3000cpm/fmol) and the reaction mixture was further incubated for 20 min at 37 °C. The reaction was quenched by adding 5 μl of loading buffer containing 50 mM EDTA, 0.5% SDS, 0.1% xylene cyanol, 0.1% bromophenol blue and 50% glycerol. The reaction products were analyzed by gel electrophoresis using a 12% polyacrylamide gel.

2) Stopped-flow fluorescence measurements: A stopped-flow FRET assay was used for measuring the unwinding kinetic rate constant of BLM, using doubly labeled DNA substrates, with fluorescein and hexachlorofluorescein as a donor and acceptor, respectively^29^,^30^. The set-up and kinetic data analysis were described in Liu *et al*.^30^. The standard reaction was performed with 4 nM DNA substrate and 60 nM protein in 25 mM Tris-HCl, pH 7.5, 50 mM NaCl, 2 mM MgCl_2_, 1 mM DTT at 37 °C.

### DNA-binding assay

The influence of Cd^2+^ on the DNA-binding activity of BLM was assayed by measuring the steady-state fluorescence anisotropy parameter^56^,^57^,^58^, using a Beacon 2000 polarization instrument (PanVera, Madison, Wi), equipped with a temperature-controlled cuvette, according to Xu *et al*.^31^. Briefly, the 3′-fluorescein-labeled DNA, free in solution and bound to BLM, are characterized by fast (low anisotropy value) and slow (high anisotropy value) rotational diffusion, respectively. The relative change in the anisotropy value allows the calculation of the fractional saturation function. Recombinant RecQ^*E.coli*^, BLM^full-length^ or BLM^642–1290^ were pre-incubated for 10 min at 25 °C with increasing concentrations of CdCl_2_ in a Tris-HCl buffer (50 mM, pH 8.0, 50 mM NaCl, 1 mM DTT) before addition of the 3′-fluorescein-labeled double- or single-stranded DNA (5 nM in a total volume of 150 μl). Fluorescence anisotropy was measured under real-time condition (steady-state fluorescence anisotropy values were recorded every 8s). The effect of Cd^2+^ on the fractional saturation function (A) (also called relative DNA-binding activity) was calculated using equation 2:


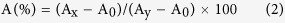


where A_x_ and A_y_ represent the fluorescence anisotropy values for a given concentration of protein in the presence or absence of CdCl_2_, respectively. A_0_ represents the anisotropy value characterizing the fluorescently labeled DNA alone.

## Additional Information

**How to cite this article**: Qin, W. *et al*. Mechanistic insight into cadmium-induced inactivation of the Bloom protein. *Sci. Rep*. **6**, 26225; doi: 10.1038/srep26225 (2016).

## Supplementary Material

Supplementary Information

## Figures and Tables

**Table 1:** Experimentally determined IC_50_ values characterizing inhibitions of ATPase and DNA-binding activities by Cd^2+^.

Activity	Determined IC_50_ values (μM)		
	BLM^642–1290^	BLM^full-length^	RecQ^*E.coli*^
ATPase	7.3	6.7	65
DNA-binding			
ssDNA (18-nt)	9.22	6.67	41.71
ssDNA (24-nt)	10.22	6.63	40.22
ssDNA (40-nt)	9.98	6.64	39.17
dsDNA (24-bp)	8.17	6.84	44.12
