# Supplementary material for: Mechanistic insight into cadmium-induced inactivation of the Bloom protein
Source: Sci Rep. 2016 May 19;6:26225. doi: 10.1038/srep26225 (PMC4872126; doi:10.1038/srep26225)
Supplement: Supplementary Information [file srep26225-s1.doc]

**Supporting Information**

**Mechanistic insight into cadmium-induced inactivation of the Bloom protein**

Wei QIN^1§^, Nicolas BAZEILLE^2§^, Etienne HENRY^2^, Bo ZHANG^1^, Eric DEPREZ^2^*, Xu-Guang XI^1,2^*

^1^College of Life Sciences, Northwest A&F University, Yangling, Shaanxi 712100, China

^2^LBPA, CNRS UMR8113, IDA FR3242, ENS Cachan, Université Paris-Saclay, 94235 Cachan, France

*Corresponding authors: email: [xxi01@ens-cachan.fr](mailto:xxi01@ens-cachan.fr); [deprez@lbpa.ens-cachan.fr](mailto:deprez@lbpa.ens-cachan.fr)

^§^These authors contributed equally to this work


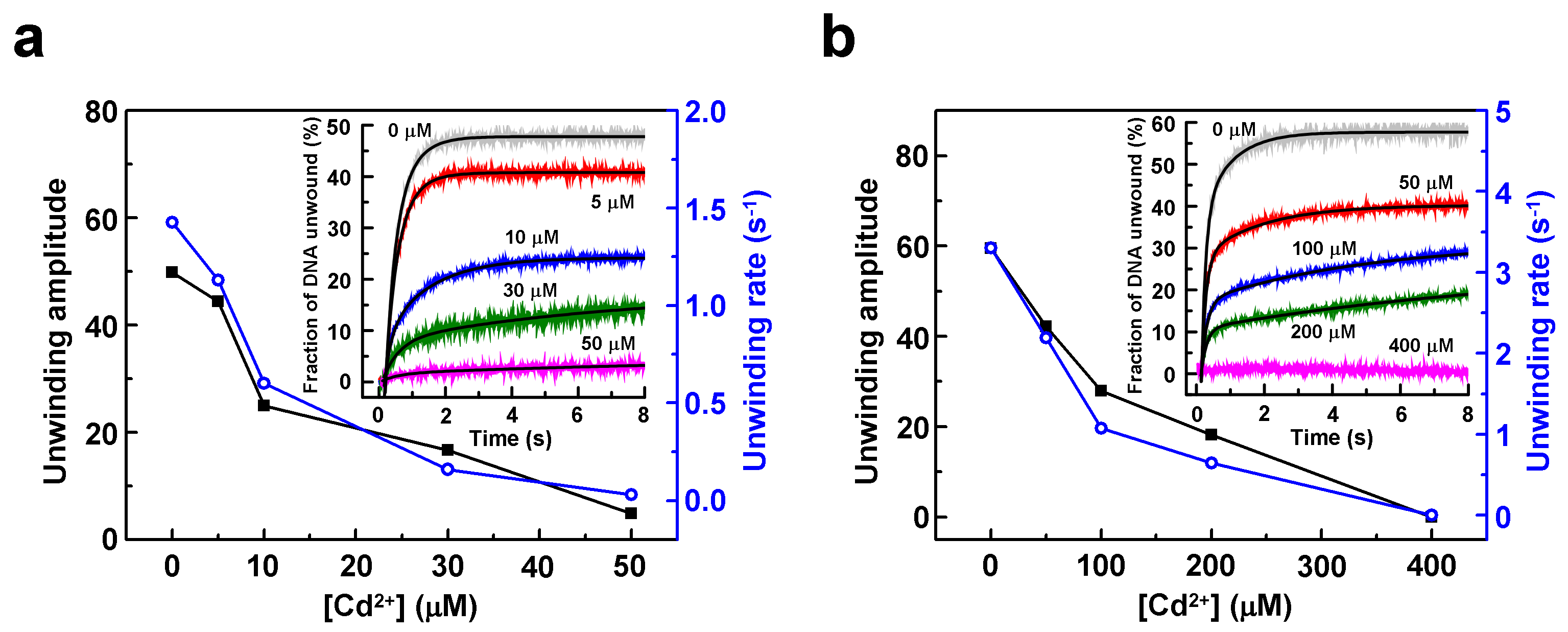
**Figure S1**: Effect of Cd^2+^ on DNA unwinding activities of BLM^full-length^ (a) and RecQ*^E.coli^* (b) as measured by stopped-flow FRET assay. Each plot shows the dependence of the kinetic rate constant and reaction amplitude as a function of CdCl_2_ concentration. Proteins (60nM) were first preincubated with varying concentrations of CdCl_2_ for 5min at 25°C. The DNA substrate (16-bp duplex with a 20-nt 3’ tail) was added into the reaction mixture at a final concentration of 4nM, and the reaction was initiated by rapid mixing with 1mM ATP. Insert: typical kinetics for DNA unwinding in the presence of various CdCl_2_ concentrations. Reactions were performed in Tris-HCl buffer (25mM, pH 7.5) supplemented with 50mM NaCl, 2mM MgCl_2_ and 1mM DTT at 37°C. The response of BLM^642-1290^ to Cd^2+^ (explicitly shown in Fig. 6a) was similar to the one observed with BLM^full-length^.


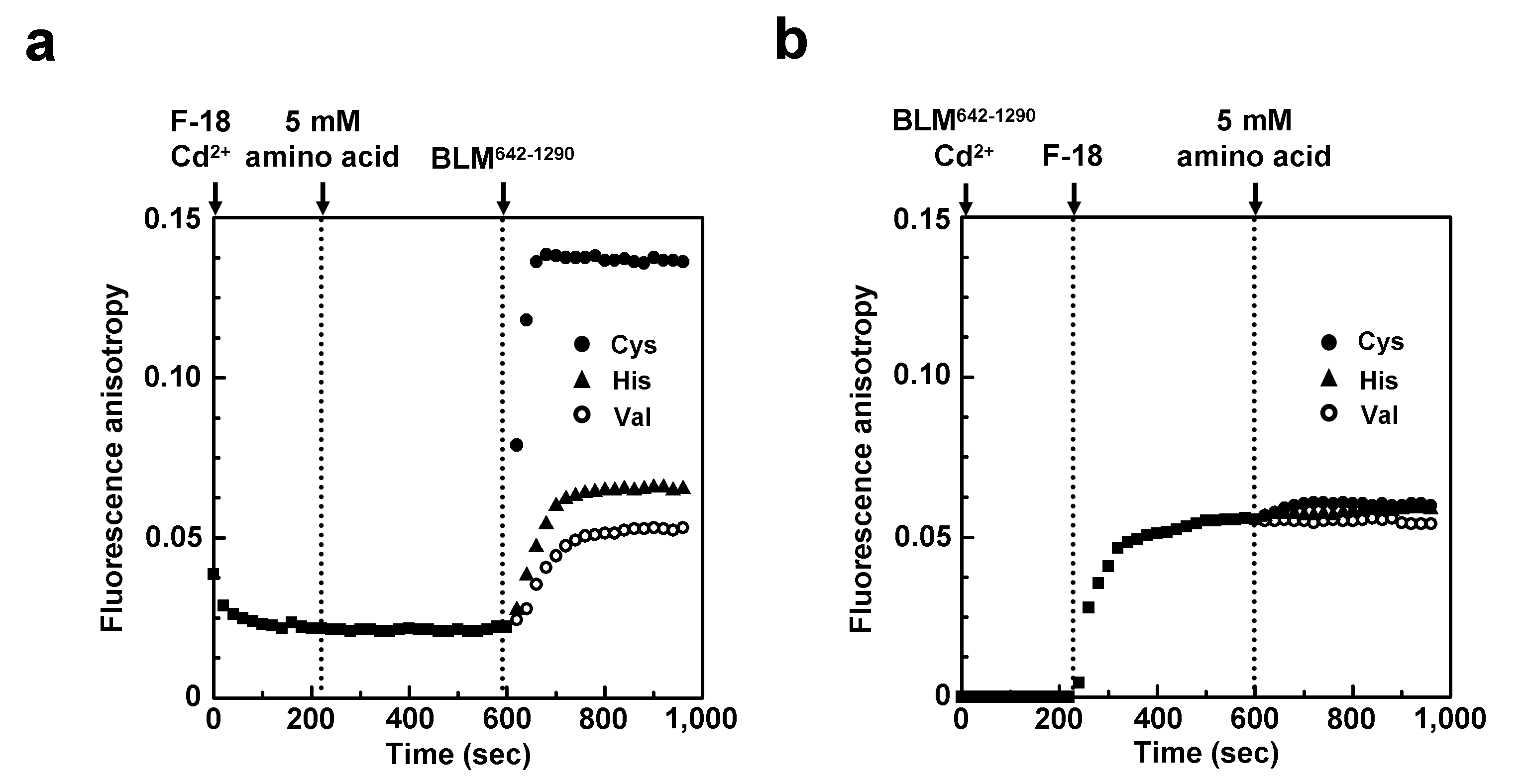
**Figure S2**: Effect of amino acids on the Cd^2+^-mediated inhibition of the DNA-binding activity of BLM^642-1290^. BLM^642-1290^ (200nM) was either added after pre-incubation of 10nM 3’-fluorescein-labeled 18-mer ssDNA (F-18), CdCl_2_ (100µM) and amino acids (5mM) (panel a) or pre-incubated with CdCl_2_ before addition of F-18 and amino acids (panel b). The steady-state fluorescence anisotropy was then monitored at 25°C as a function of time.


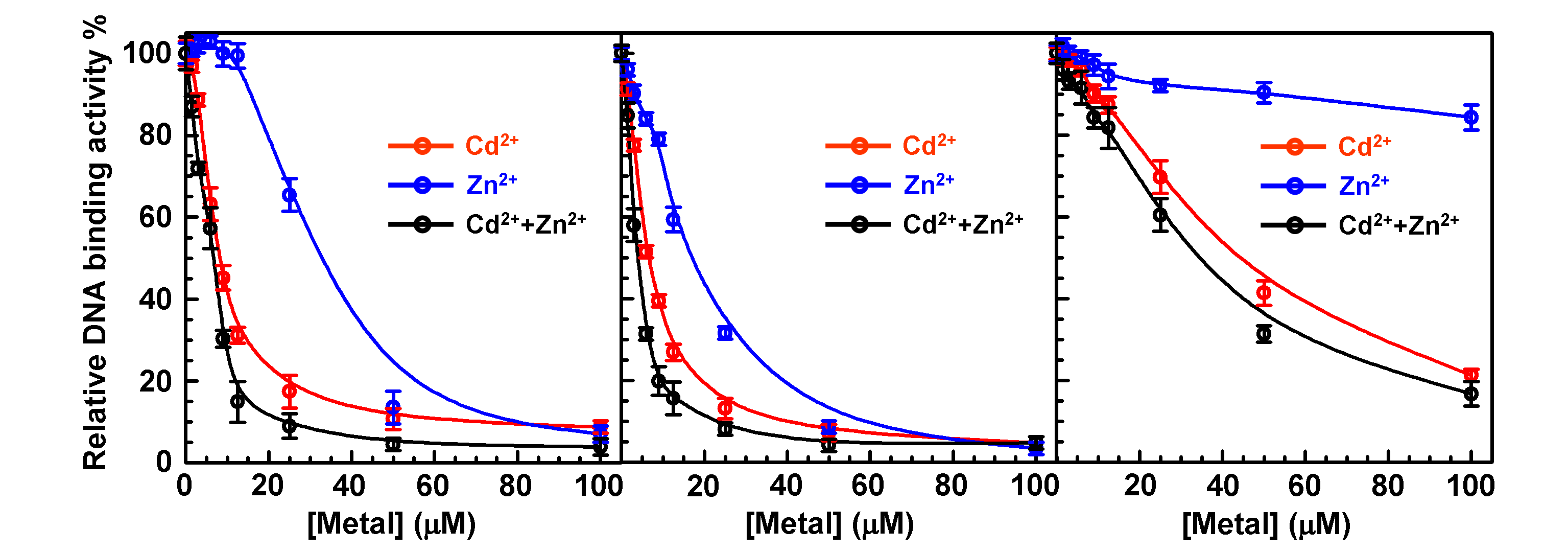
**Figure S3**: Effect of Cd^2+^, Zn^2+^ or a combination of both cations on the DNA-binding activities of BLM^642−1290^ (left), BLM^full-length^ (middle) and RecQ*^E.coli^* (right). Increasing concentrations of Cd^2+^, Zn^2+^ or Cd^2+^/Zn^2+^ were added on pre-formed helicase/DNA complexes. The concentration relative to Cd^2+^/Zn^2+^ combination, indicated on the x-axis, corresponds to the total concentration of cations where [Cd^2+^] = [Zn^2+^]. The fluorescence anisotropy was measured at 25°C using 200nM protein and 5nM F-24 oligonucleotide as described in Methods. The relative DNA-binding activity was calculated according to Eq. 2.


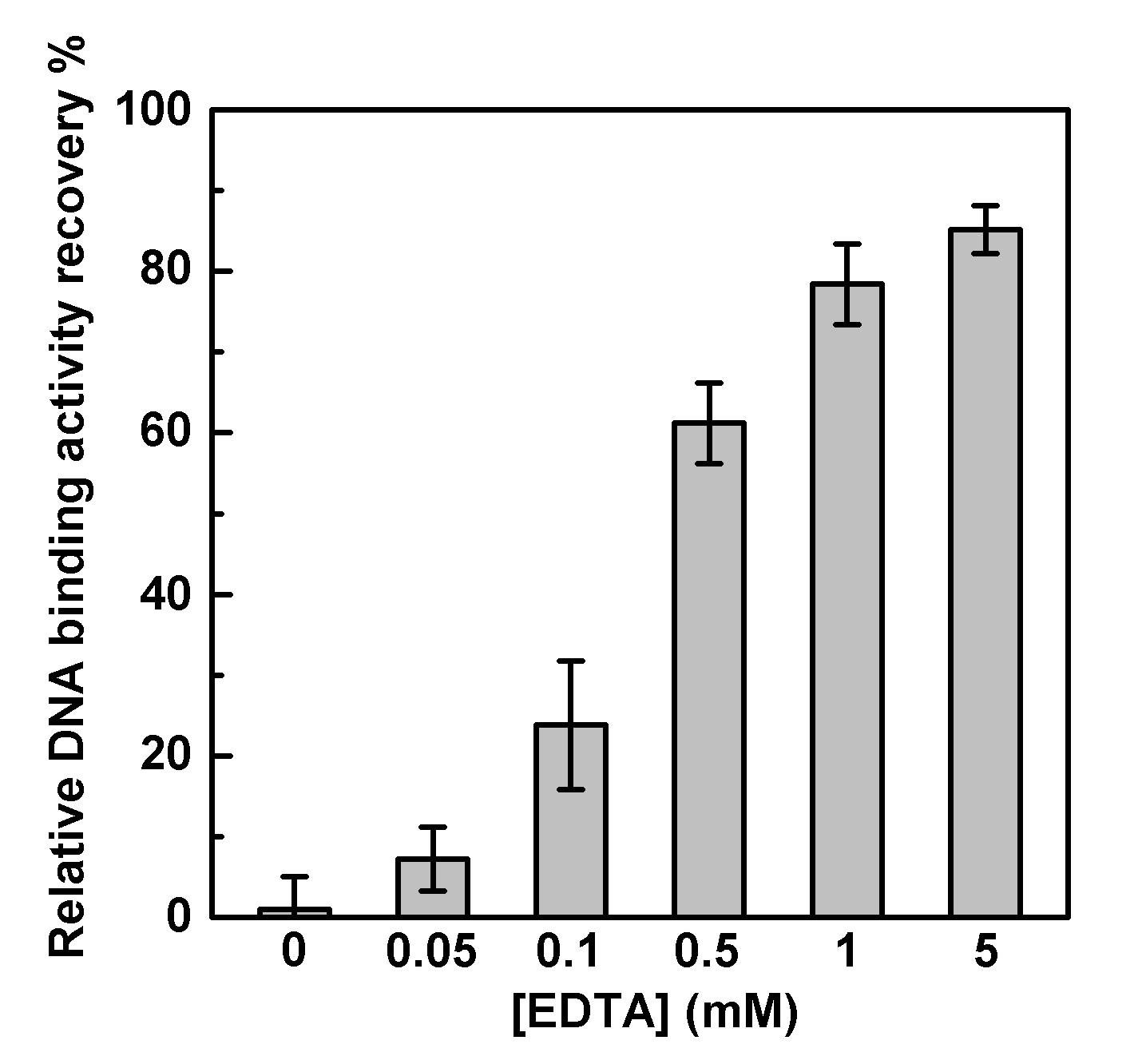
**Figure S4**: Relative recovery of the DNA-binding activity of BLM^642-1290^ as a function of EDTA concentration after Cd^2+^-induced dissociation. Concentrations of protein, ssDNA (F-18) and Cd^2+^ were 200nM, 5nM and 50µM, respectively. The relative DNA-binding activity was determined in a Tris-HCl buffer (50mM, pH 8.0, 50mM NaCl, 1mM DTT) according to Eq. 2.


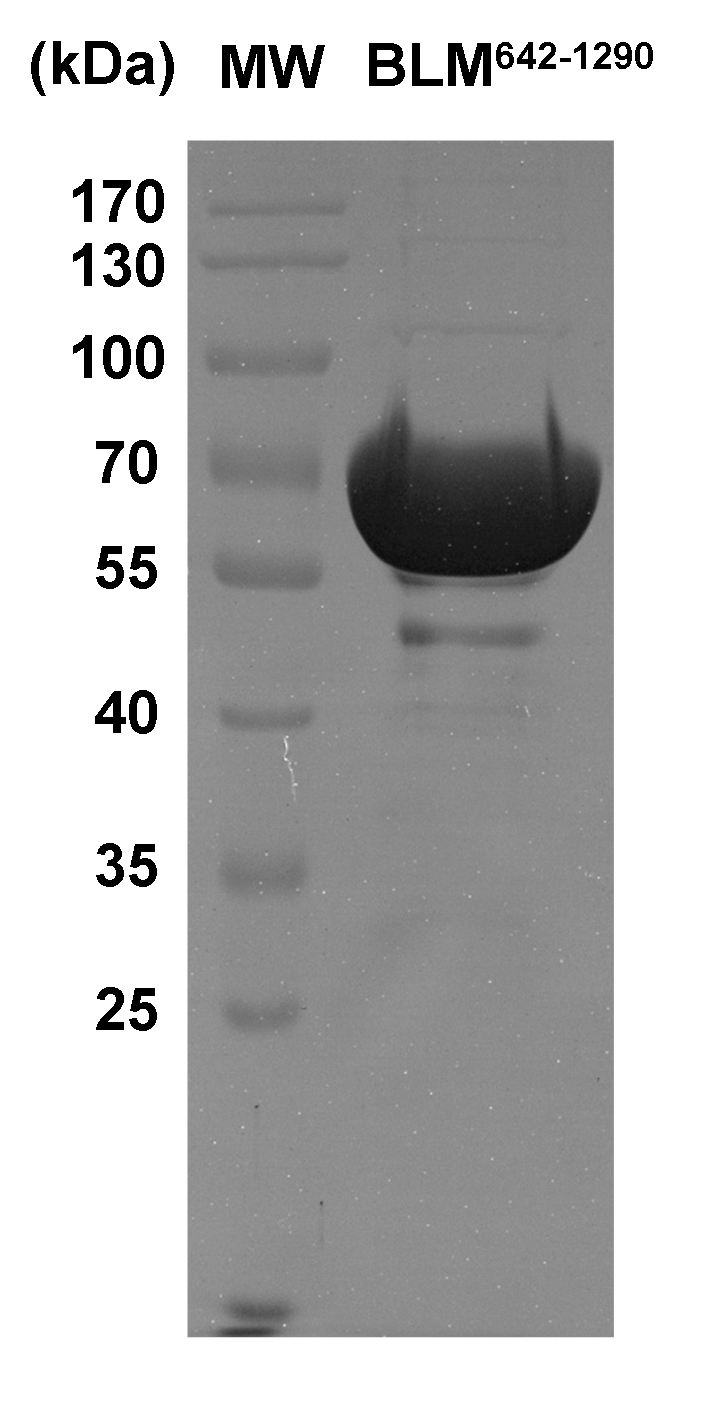
**Figure S5**: SDS-PAGE analysis of the purified recombinant BLM^642-1290^ protein. The gel (10% acrylamide) was stained using Coomassie brilliant blue R250. The amount of BLM^642-1290^ protein loaded on the gel was 12µg.

**Table S1**. Structures of the DNA substrates used for the measurement of helicase, ATPase and DNA-binding activities.

| Substrates | DNA sequence |
| --- | --- |
| Radioactive DNA unwinding assay: |  |
| 25-bp duplex with 19-nt 3’-ssDNA tail | 5’-GCACTGGCCGTCGTTTTACGGTCGTGACTGGGAAAACCCTGGCG-3’ |
|  | 3’-AACTTTTTTTTTTCCCCAACCAGCACTGACCCTTTTGGGACCGC-5’ |
| Stopped-flow FRET DNA unwinding assay: |  |
| 16-bp duplex with 20-nt 3’-ssDNA tail | 5’-CTCTGCTCGACGGATT-F^a^-3’ |
|  | 5’-HF^b^-AATCCGTCGAGCAGAGtttttttttttttttttttt-3’ |
| ATPase activity assay: | 5’-AACCAACAACAACAACAACAACAAC-3’ |
| Fluorescence anisotropy-based DNA binding assay: |  |
| F-18 | 5’-GCCTCGCTGCCGTCGCCA-F-3’ |
| F-24 | 5’-GCCCTGCTGCCGACCAACGAAGGT-F-3’ |
|  | 3’-CGGGACGACGGCTGGTTGCTTCCA-5’ |
| F-40 | 5’-GCCCTGCTGCCGACCAACGATGGTTACATTCCCGCTGCTG-F-3’ |

^a^F, fluorescein

^b^HF, hexachlorofluorescein

**Table S2. Spatial distribution of cysteine residues in BLM^642-1290^ and RecQ*^E. coli^*.**

| **BLM^642-1290^** | | **RecQ*^E. coli^*** | |
| --- | --- | --- | --- |
| **Cys position** | **Cys distribution** | **Cys position** | **Cys distribution** |
| 685* | Inner | 43* | Inner |
| 698* | Inner | 56* | Inner |
| 704 | Surface | 94 | Surface |
| 771 | Surface | 111 | Inner |
| 799* | Surface | 150* | Surface |
| 878 | Inner |  |  |
| 895* | Inner | 243* | Inner |
| 901 | Inner |  |  |
| 940 | Surface |  |  |
| 944 | Inner |  |  |
| 989 | Inner |  |  |
| 1030 | Surface | 351 | Surface |
| 1036* (ZFD) | Surface | 380* (ZFD) | Inner |
| 1055* (ZFD) | Inner | 397* (ZFD) | Surface |
| 1063* (ZFD) | Inner | 400* (ZFD) | Inner |
| 1066* (ZFD) | Surface | 403* (ZFD) | Surface |
| 1067 | Surface |  |  |
| 1218 | Surface |  |  |
| 1226 | Inner |  |  |

The spatial conformation distribution of cysteine residues of BLM^642-1290^ and RecQ*^E. coli^* were analyzed by PyMol using the X-ray three dimensional structures of BLM^642-1290^ (PDB file, 4CGZ) and RecQ*^E. coli^* (PDB file, 1OYW). Cys labeled with stars represent conserved residues at the primary sequence and 3D structural levels.
